# Rice husk silica blended fillers for engine mount application

**DOI:** 10.1038/s41598-024-53742-5

**Published:** 2024-02-06

**Authors:** Paschal A. Ubi, Nuhu A. Ademoh, Esther N. Anosike-Francis, Abdulrahman A. Salawu, Adekunle A. Adeleke, Uzoma G. Okoro, Aliyu A. Abdullahi, Frederick Ngolemasango

**Affiliations:** 1 Department of Mechanical Engineering, University of Calabar, PMB 1115, Calabar, Cross River State Nigeria; 2 Department of Mechanical Engineering, Federal University of Technology Minna, PMB 65, Minna, Niger State Nigeria; 3 Department of Mechanical Engineering, Nile University of Nigeria, Plot 681, Cadastral Zone C, Airport Road, Jabi, Abuja, Abuja Federal Capital Territory Nigeria; 4 Department of Materials and Metallurgical Engineering, Federal University of Technology Minna, PMB 65, Minna, Niger State Nigeria; 5 Department of Chemistry, University of Buea, Buea, Cameroon; 6 DTR/VMS, Aintree Avenue, Trowbridge, Wiltshire, UK

**Keywords:** Engineering, Materials science

## Abstract

The functional properties of engine mounts largely depend on the rubber compound formulation. This study proposes the use of rice husk–derived silica (RHS) blended with carbon black (N772) as an effective and environmentally friendly substitute for fillers used in rubber engine mounts (REMs). CV-60 natural rubber was filled with the blended fillers at various ratios, and their compatibility for use as rubber engine mounts (REMs) was assessed. Grey Relational Analysis was utilised to determine the optimal blend loading levels for use in rubber engine mounts, resulting in 40 phr of N772 and 20 phr of RHS cured at 130 °C and 2.5 MPa for 20 min. The developed REMs and conventional REMs had low vibration data variation during the performance assessment. Their resonance transmissibility was 5.03 and 3.74, corresponding to natural frequencies of 24.27 Hz and 26.94 Hz, respectively. The RHS/N772 REMs had excellent damping characteristics and lower transmissibility in the isolation zone of the vibration isolation curve, which is outside of the resonant frequency region. The efficiency curves showed that the blended fillers are a better and more effective material for REMs at all frequencies, balancing static deflection and vibration isolation.

## Introduction

In today’s society, utilising sustainable and eco-friendly resources is expanding and simultaneously dominating both current and emerging markets. Agricultural residues are anticipated to be a growing engineering material not just considering the technological aspect, but also taking into account the social and environmental aspects., as indicated by a variety of contemporary economic, ecological, and production policies established by governments^1,2^. This has led to continuous investigations and the application of bio-based sustainable materials to replace the prevalent materials utilised in the field of engineering manufacture over the period of the last five decades^2–4^. These bio-based materials have been proven to be feasible and successful alternatives to conventional materials when it comes to cost reduction, savings in energy, the impact on the environment and even manufacturing economics^5–8^.

Challenges related to waste management and the utilisation of unsustainable and environmentally detrimental resources in the production of engineering products have necessitated research into more effective ways to improve sustainability. This is obvious in the United Nations' Sustainable Development Goals (SDGs), which are being implemented from 2016 to 2030^9^. The increased investment interest in rice production to meet the current demand for the commodity corresponds to a rise in rice husks, which, if not properly handled, can pose significant environmental and health risks^10^. Rice husks, when burned on farmlands, pose enormous threats to the environment from carbon dioxide emissions into the atmosphere and the onset of silicosis, a currently incurable disease. Many areas of application of rice husks have been investigated and developed, including low cost building materials^11^, cement for building construction, as reinforcement in local mud bricks/earth houses, biomass for energy production, ceramics for home and industrial purposes, dye manufacture, electronic chips^10^ and batteries^12–14^.

The functional characteristics of products made of rubber are primarily determined by the formulation of the compound, which includes the cure system and the type of filler employed. Carbon black and commercial silica, both of which are non-biodegradable fillers, have been the primary focus of elastomeric product development for many years. A study was carried out on the partial replacement of fillers employed in natural rubber composites for use in tyres^15^. The study utilised carbon black N134 grade (CB) and commercial silica (SiO_2_) while maintaining a consistent filler content of 55 parts per hundred rubber (phr) and varying the composition of the individual fillers ratios in the blend. Results from study^15^ established that SiO_2_/CB blend yields better composites performance having improved abrasion resistance, wet grip with bound rubber content (BRC) and other mechanical characteristics showing an optimum at 45/10 phr silica–carbon black blend filler ratio. Several researchers have stated that rice husk contains a high percentage of silica, and it is generally known that silica is an important filler in rubber manufacturing. Although the utilisation of carbon black blended with commercial silica has been extensively researched, the use of rice husk-derived silica as a blend with carbon black and its application in vibration isolators are yet to be considered. As a result, this study investigated the possibility of using silica extracted from rice husks as a blend with N772 and, subsequently, its suitability for usage in vibration isolation application domains such as rubber engine mounts.

Vibration and, consequently, the discomfort experienced by vehicle occupants are mostly caused by the dynamic forces acting on vehicles during operation^16,17^. Researchers^18^ have stated that despite the remarkable vibration isolation characteristics of current engine mounts, further enhancements are required to optimise the performance of the rubber engine mount system, notably by including bio-based materials in its manufacture. The rubbers used as the basic material for engine mounts are typically reinforced with carbon black, commercial silica, or a blend of both. Global carbon black output is estimated at 8 million metric tonnes per year, with around 92% of that quantity utilised in the manufacture of rubber products^19^. These fillers are non-renewable, consume a substantial amount of energy during processing, are not sustainable, contribute significantly to global carbon emissions, and are rare and expensive^20–22^. In addition to generating serious health problems such as cancer^23^, the production of these conventional fillers causes irreversible environmental damage. Commercial silica, which is presently used as a replacement for carbon black, especially in tyres and rubber mount compounds, generates a large quantity of wastewater, resulting in a high level of environmental contamination, and consumes a significant amount of energy during its processing phase^4,24^. These difficulties have paved the way for the development of emerging environmentally friendly alternatives, such as the utilisation of silica obtained from rice husk^25,26^. Approximately 2.4 tonnes of carbon dioxide (CO_2_) are generated by the partial burning of heavy hydrocarbons during the production of 1 tonne of carbon black^27,28^. Moreover, investigations^29^ have revealed that 1 tonne of Rice Husk Silica lowers emissions of carbon dioxide by 1.29 tonnes, which corresponds to a 50 percent reduction in CO_2_ emissions. The integration of rice husk silica into natural rubber compounds, whether as single fillers or as blends, promotes energy savings and a substantial reduction in environmental concerns associated with the disposal of rice husk and carbon emissions^26^. Studies^30^ have posited that the combination of silica and carbon black gives the combined benefits of the separate fillers in rubber composites, thereby promoting the use of blended fillers as opposed to individual fillers.

It is expected that biobased alternatives would be commercially viable, sustainable, and processed using environmentally sustainable methods^22,31–33^. Therefore, a need exists for studies on sustainable, high-performance bio-based fillers that will make a significant contribution to resolving growing environmental concerns while permitting the final products to compete favourably with those from existing pairs and improving the attributes of conventional rubber engine mounts. The addition of bio-based filler particles like rice husk silica to rubber compounds not only enhances the strength, reliability, and performance of the final products, but also significantly minimises the material and processing costs^19,26,34^, generating over 4% energy savings in comparison to tyre compounds developed using carbon black^35^, and helps to resolve environmental and health-related challenges. Dominic et al.^22^ in a study examined the effect of combining carbon black with rice husk nano-cellulose to reinforce natural rubber. They found that rice husk nano-cellulose was effective in reducing rolling resistance, an essential characteristic in the production of tyres.

The use of rubber engine mounts entails contradictory requirements and necessitates a compromise for sustained performance, specifically in terms of stiffness and damping characteristics. Engine mounts which support static and dynamic loads alongside damping vibrations have to be rigid and simultaneously possess a high damping property, which promotes the safety of vehicles' occupants during driving and enhances their comfort. Material and design are the two most critical aspects in determining an engine mount's energy absorption behaviour^36,37^. For natural rubber composites to be of tremendous value to the industry, it is necessary to determine and comprehend the pertinent variables and material mixes, such as the kinds of filler particles, the filler proportions, especially in the case of hybrids or blends, the additives, and the optimum process conditions^38,39^. This research centred on the material element and production characteristics of the load-bearing portion of rubber engine mounts, using rice husk silica blended with N772 carbon black as the reinforcement in natural rubber while conforming to the existing mount design. Bio-based blended composites can be employed as a suitable replacement for elastomeric parts of rubber engine mounts to meet the sustainability requirements of engineering materials, improve the quality of products, and maintain the minimum cost of production in terms of energy and funds while maintaining the established excellent performance requirements of rubber engine mounts.

## Method

### Preparation of rice husk silica

**Figure 1:**
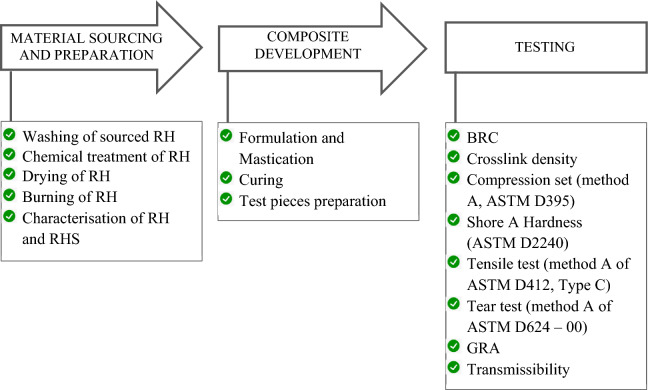
The process adopted in the study.

Rice husks gathered from a rice mill in Zaria, Nigeria, were used in this study. After being thoroughly cleaned with water, the rice husks were spread out to dry. A 0.4 M HCl solution was made and used to treat the rice husks to further improve the purity of the silica output and reduce contaminants. The concentration of the hydrochloric acid was 35.4%, its density was 1.18 g/ml, and its molar mass was 36.5 g/mol. 100 g of rice husks were added to 1 L of the prepared HCl acid solution for each batch, which was then heated using a heating mantle to 100 °C for 40 min. Following the heating time, the rice husks were removed from the solution and thoroughly rinsed with water to remove any HCl traces before being dried in an electric oven at 100 °C for five hours. High-purity rice husks silica was obtained by combusting the treated, oven-dried rice husks for six hours at 600 °C in an electric furnace. Figure 1 presents a schematic of the method adopted in the study.

### Design of experiment

Table 1 presents the factors and their respective levels as determined and developed for this study. As shown in Table 2, the RHS and N772 filler blending ratios were computed by applying a 2-factor, 5-level full factorial design of experiment. The minimum filler loading of 50 phr was determined based on the findings of various studies which revealed that filler loading of vibration isolators is typically within 50 to 90 phr to obtain outstanding mechanical and physical properties of the composites^40,41^.

**Table 1:** Fillers and their corresponding levels.

Variable	Unit	Level 1	Level 2	Level 3	Level 4	Level 5
N772	Phr	40	50	60	70	80
RHS	Phr	10	20	30	40	50

**Table 2:** Full factorial DOE for the RHS/N772 blend.

Composite ID	StdOrder	RunOrder	PtType	Blocks	N772	RHS
C40R10	1	1	1	1	40	10
C40R20	2	2	1	1	40	20
C40R30	3	3	1	1	40	30
C40R40	4	4	1	1	40	40
C40R50	5	5	1	1	40	50
C50R10	6	6	1	1	50	10
C50R20	7	7	1	1	50	20
C50R30	8	8	1	1	50	30
C50R40	9	9	1	1	50	40
C50R50	10	10	1	1	50	50
C60R10	11	11	1	1	60	10
C60R20	12	12	1	1	60	20
C60R30	13	13	1	1	60	30
C60R40	14	14	1	1	60	40
C60R50	15	15	1	1	60	50
C70R10	16	16	1	1	70	10
C70R20	17	17	1	1	70	20
C70R30	18	18	1	1	70	30
C70R40	19	19	1	1	70	40
C70R50	20	20	1	1	70	50
C80R10	21	21	1	1	80	10
C80R20	22	22	1	1	80	20
C80R30	23	23	1	1	80	30
C80R40	24	24	1	1	80	40
C80R50	25	25	1	1	80	50

### Compounding

**Figure 2:**
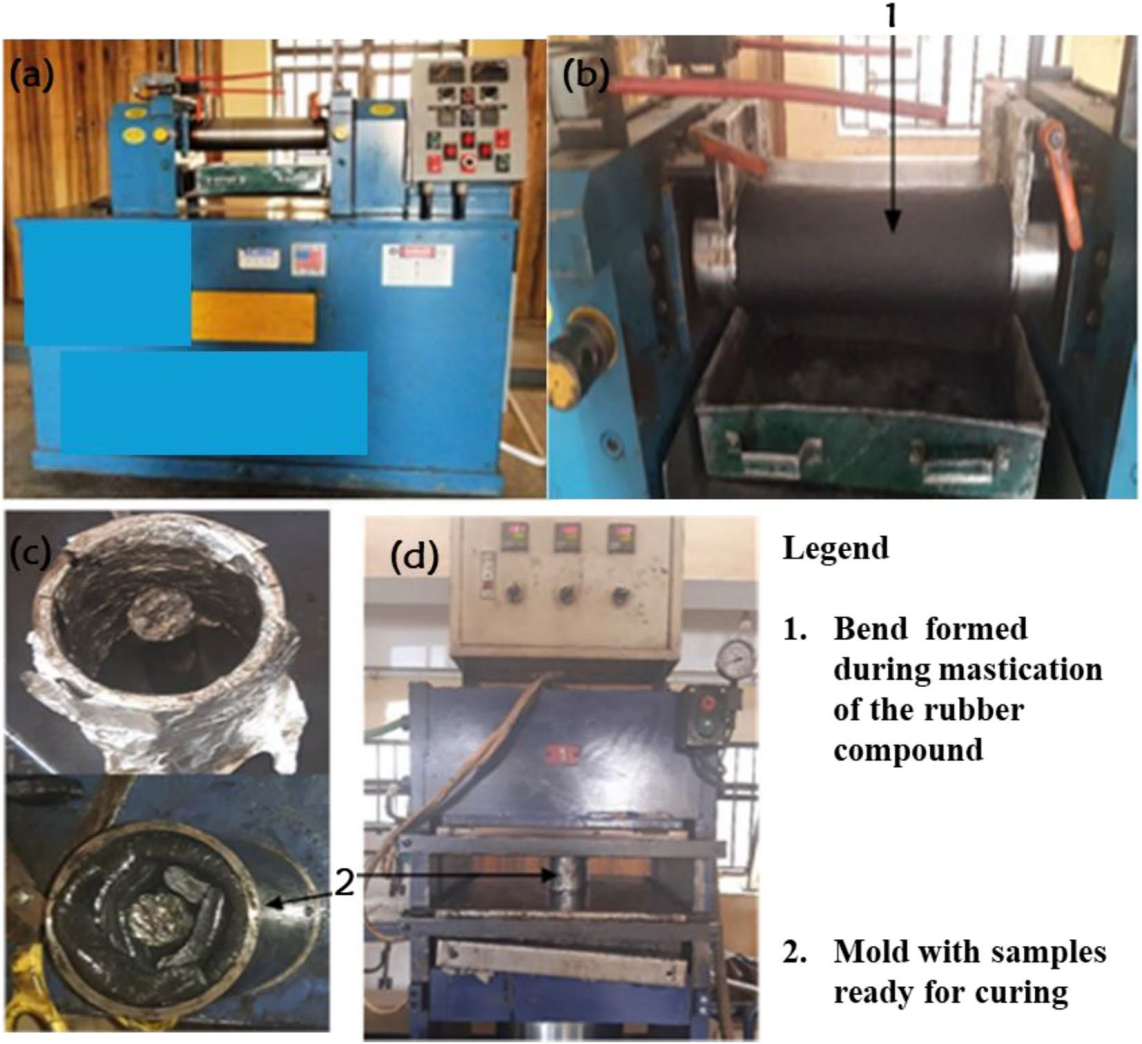
(**a**) Two-roll mill used for compounding (**b**) Compounding in progress (**c**) loading of compounded composite (**d**) Compression molding machine used for curing.

Conforming to ASTM D3182-07, the fillers and CV-60 natural rubber were compounded on a two-roll mill manufactured by Reliable Rubber and Plastic Company, New Jersey (See Fig. 2). The rollers were heated to 70 °C and had a speed ratio of 1:1.25, rotating at 24 rpm. Zinc oxide was employed as an activator, while stearic acid served as a processing aid and an accelerator activator playing a vital role in the vulcanization of the compound. The anti-oxidants used during the compounding are Vulcanox 4020 and TMQ (2,2,4-Trimethyl-1,2-dyhydroquinoline) while the accelerators used were TBBS (N-tert-butyl-2-benzothiazolesulfenamide), and TMTD (Tetramethylthiuram disulphide). Sulphur was utilised as the curing agent. Due to compatibility concerns connected with silica in natural rubber, the coupling agent silane TESPT (3-(Triethoxysiliyl) propyl tetrasulphide) was employed. All masticated compounds were cured for 20 min using a compression moulding machine with plates set at 130 °C at a pressure of 2.5 MPa. Table 3 presents the information on the composition of rubber mixtures, as well as the order and time of incorporation of the components.

**Table 3:** Compounding sequence for all samples.

Stage	Time of inclusion (min)	Operation
1	0	Rubber is cut into pieces, incorporated, and masticated
2	2	ZnO, Stearic acid, 6PPD, TMQ, and accelerators are added
3	4	Half of the filler and processing aid are incorporated
4	6	Incorporation of the remaining filler and remaining processing aid
5	8	Half of the sulphur formulation introduced to the mix on the roll mill
6	10	The remainder of sulphur is added
7	12	Material is formed, cut and re-banded 6 times
8	14	The compound is sheeted out, dumped, labelled and conditioned

### Testing

The tests conducted in this study included specific parameters associated with standard tests for rubber mounts, as gathered from existing literature and standards by the Automotive Industry Standards AIS-079, Society of Automotive Engineers SAE J1085/J1636, and various ASTM standards for testing elastomers.

### Bound rubber content (BRC) and crosslink density

The bound rubber content was conducted tso assess the surface activity of the blended fillers and matrix in the composites. The BRC provides data on the quantity of natural rubber that cannot be removed by toluene, which quantifies the matrix's adsorption on the surface of blended fillers. A composite with a high BRC demonstrates a stronger and superior filler-matrix interaction. The bound rubber content is computed using the formula presented in Eq. (1).

1 $$ {\text{BRC }}\left( \% \right) \, = \frac{{W_{fr} - W\left[ {\frac{{m_{f} }}{{m_{f} + m_{r} }}} \right]}}{{W\left[ {\frac{{m_{r} }}{{m_{f} + m_{r} }}} \right]}} \times 100 $$

where *W*_*fr*_ = weight of the blended filler and rubber after extraction, *W* = weight of the compound before extraction, *m*_*f*_ = weight of the blended filler in the compound, *m*_*r*_ = weight of the CV-60 rubber in the compound.

The crosslink density (Vcd) was calculated following the ASTM D471-16a standard utilising the Flory-Rehner formulas presented in Eqs. (2) to (4).

2 $$ V_{cd} = \frac{1}{{2M_{c} }} = - \frac{{In\left( {1 - V_{r} } \right) + V_{r} + \chi V_{r}^{2} }}{{2\rho_{r} V_{s} \left( {\sqrt[3]{{V_{r} }} - \frac{{V_{r} }}{2}} \right)}} $$

3 $$ V_{r} = \frac{{\frac{{W_{1} - W_{f} }}{{\rho_{r} }}}}{{\frac{{W_{1} - W_{f} }}{{\rho_{r} }} + \frac{{W_{2} - W_{1} }}{{\rho_{s} }}}} $$

4 $$ \chi = \beta + \frac{{V_{s} }}{RT}\left( {\sigma_{p} - \sigma_{s} } \right)^{2} $$

where *V*_*cd*_ = crosslink density (mol/cm^3^), *Mc* = average molecular weight of the rubber between crosslinks (g/mol), *V*_*r*_ = volume fraction of the swelled rubber gotten from the densities and mass of rubber and toluene, $\chi$ = Flory Huggins rubber-toluene interaction parameter, *V*_*s*_ = molar volume of toluene (106.27 cm^3^/mol), $\rho_{r}$ = density of the natural rubber (g/cm^3^), $\rho_{s}$ = density of toluene (g/cm^3^), *W*_*1*_ = weight of the RHS/N772-NR composite before swelling (g), *W*_*2*_ = weight of the RHS/N772-NR composite after swelling (g), *W*_*f*_ = weight of the fillers (g), $\beta$ = 0.34 (lattice constant for polymer–solvent blends), *R* = gas constant, *T* = absolute temperature in kelvin (293.15 K), $\sigma_{p}$ = solubility parameter of elastomer (16.7 MPa^1/2^ for natural rubber), $\sigma_{s}$ = solubility parameter of toluene (18.0 MPa^1/2^).

### Mechanical tests

Compression set (method A, ASTM D395 standard), Shore A Hardness (ASTM D2240), Tensile test (ASTM D412 standard, method A–Type C), and premised on the tensile stress at break and Tear test (method A of ASTM D624–00 standard) are the mechanical tests conducted in this study.

### Grey relational analysis (GRA)

Grey Relational Analysis was employed to identify the optimal RHS/N772 blend by decreasing the multi-response parameters to a single response, hence providing the most viable option from the effect of the factors. GRA was selected as a technique among others as a result of the advantages it provides for generating quality judgments, even in complex systems with limited and, in most situations, sparse information^42,43^. For compression set and shore A hardness, a smaller-is-better quality characteristic as stated by Eq. (5) was introduced. For signal-to-noise (S/N) ratio computations of tensile strength, tensile modulus, tear strength, bound rubber content, and crosslink density, the larger-the-better quality characteristic as given by Eq. (6) was utilised.

5 $$ {\text{S}}/{\text{N }}\left( {{\text{Smaller }}\,{\text{the}}\,{\text{ better}}} \right) \, = - 10\log \frac{1}{n}\left( {\mathop \sum \limits_{i - 1}^{n} x_{i}^{2} } \right) $$

6 $$ {\text{S}}/{\text{N }}\left( {{\text{Larger}}\,{\text{ the}}\,{\text{ better}}} \right) \, = - 10\log \frac{1}{n}\left( {\mathop \sum \limits_{i - 1}^{n} \frac{1}{{x^{2} }}} \right) $$

where x = response of the factor level combination, n = number of experimental samples.

The data used in Grey Relational Analysis are usually pre-processed into quantitative indices and normalized before obtaining the deviation sequence, the Grey Relational Coefficient (GRC) and the Grey Relational Grade (GRG). To make comparisons easier, an original sequence must be converted into decimal places between 0.00 and 1.00 as part of the pre-processing of the data. Equation (7) is used to normalise the original sequence for data sequences that are anticipated to conform to higher-the-better characteristics, while Eq. (8) is used to normalise the original sequence for smaller-the-better characteristics.

7 $$ x_{i}^{*} \left( k \right) = \frac{{x_{i}^{0} \left( k \right) - min.x_{i}^{0} \left( k \right)}}{{max.x_{i}^{0} \left( k \right) - min.x_{i}^{0} \left( k \right)}} $$

8 $$ x_{i}^{*} \left( k \right) = \frac{{max.x_{i}^{0} \left( k \right) - x_{i}^{0} \left( k \right)}}{{max.x_{i}^{0} \left( k \right) - min.x_{i}^{0} \left( k \right)}} $$

where $x_{i}^{0} \left( k \right)$ represents each response value; *max*. and *min.* represents the maximum and minimum value in the data set of the response value under consideration.

The normalising sequence's values are typically subtracted from “1” to get the deviation sequence, which shows how much each response deviates from unity. The GRC computation is carried out to demonstrate the connection between an ideal and actual experimental result. The GRC is calculated using the expression in Eq. (9).

9 $$ {\text{GRC}} = \frac{{\Delta_{min} + \propto \Delta_{max} }}{{\Delta_{ij} + \propto \Delta_{max} }} $$

where $\Delta$_ij_ = deviation sequence value of the concerned response (for i = 1, 2, … , m and j = 1, 2, … , n), $\Delta$_min_ = minimum of the deviation sequence value, $\Delta$_max_ = maximum value in the deviation sequence for the concerned response, α = distinguishing coefficient which is used in expanding or compressing the GRC values.

The distinguishing coefficient (α) often assumes a value of 0.5 in most GRG computations^43^. The average of all GRC values across all rows was used to determine the GRG. By averaging similar levels from the experimental design, the factor effects that emerge for the GRG can be evaluated. All design of experiment and calculations were performed using Minitab 19 and Microsoft Excel respectively.

### Performance evaluation

**Figure 3:**
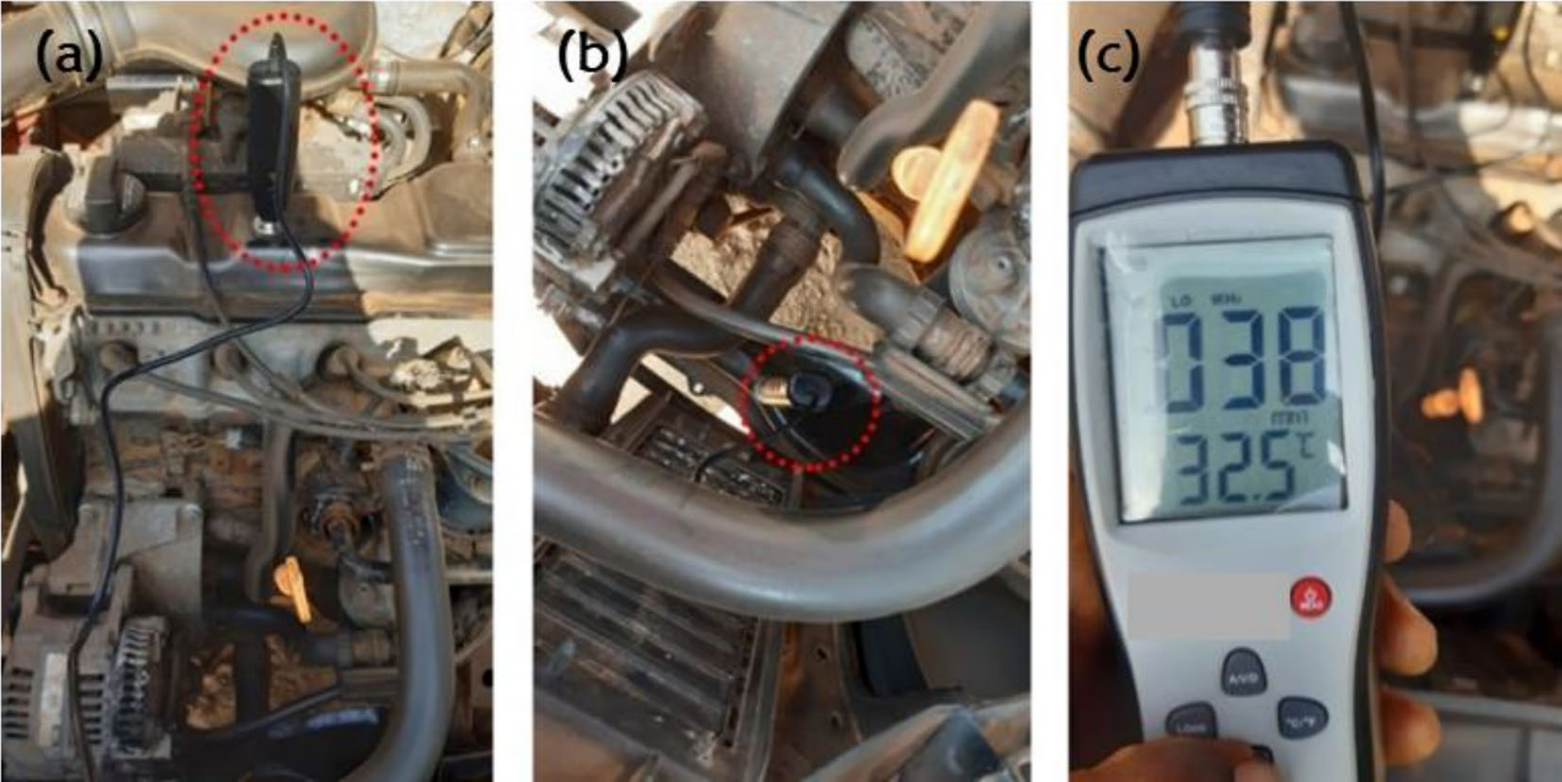
(**a**) Magnetic probe on the cylinder head (**b**) Magnetic probe on the chassis beside the front REM (**c**) Vibration meter used for measurements.

A vehicle was used to evaluate the performance of the REM produced from the RHS/N772 blend in comparison to that of an existing traditional REM. A smart sensor vibration meter (shown in Fig. 3) was used to measure the velocity, acceleration, and displacement at the cylinder head and chassis for both the traditional mounts and the produced blended RHS/N722 REM.

The Force Transmissibility (TR) and the efficiency of vibration isolation (E_ff_) were computed using the expressions in Eqs. (10) and (11) respectively.

10 $$ {\text{TR }} = \frac{{\sqrt {1 + \left( {2\zeta \beta } \right)^{2} } }}{{\sqrt {\left( {1 - \beta^{2} } \right)^{2} + \left( {2\zeta \beta } \right)^{2} } }} $$

11 $$ {\text{E}}_{{{\text{ff}}}} = \, \left( {{1 }{-}{\text{ TR}}} \right) \, \times { 1}00 $$

where ζ is the damping factor and β is the frequency ratio which was computed while adopting the expressions governing vibration systems using preliminary findings and presented in Table 4.

**Table 4:** Parameters used in the determination of the transmissibility.

Property	Prototype	Existing REM
Storage modulus, E′ (MPa)	0.7208	4.8092
Loss modulus, E′′ (MPa)	0.1455	1.3226
Force from engine mass, F (N)	1550	1550
Deflection (mm)	0.0002	0.0011
Tangent loss factor (η)	0.2019	0.2750
Loss angle (delta)	0.1992	0.2684
Complex modulus, E* (MPa)	0.7353	4.9878
Spring stiffness, k* (N/mm) @33 °C	3676.69	4534.32
Natural frequency, Wn (Hz)	24.27	26.96
Critical damping coef. (Cc) =	1524.36	1692.84
Damping factor (ζ) =	0.1015	0.1389
Damping coefficient (c) =	154.65	235.05

## Results and discussion

**Figure 4:**
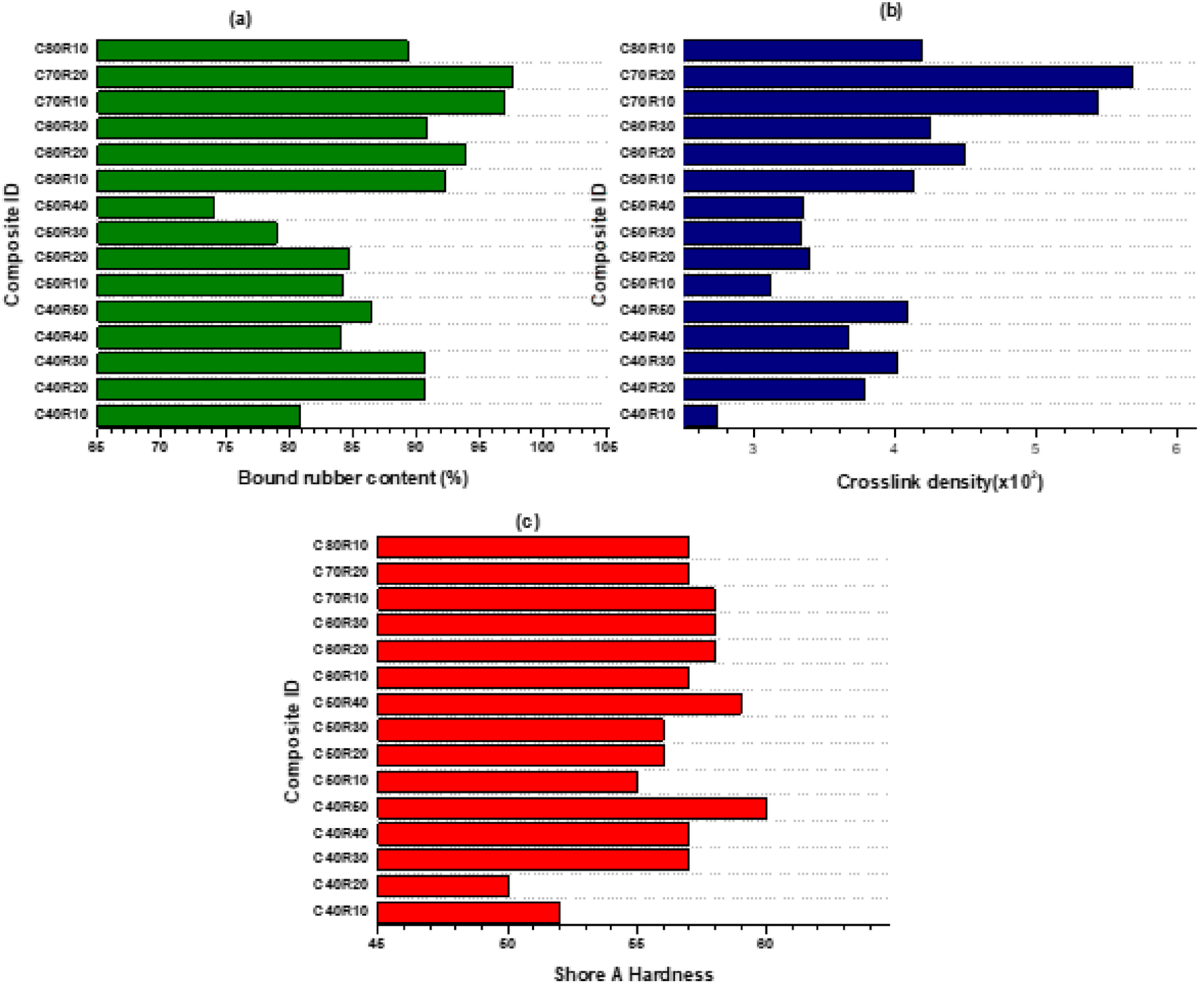
(**a**) Bound rubber content of N772/RHS filled Composites (**b**) Crosslink density of N772/RHS filled composites c. Shore A hardness of N772/RHS filled composites.

As shown in Fig. 4a, the bound rubber content of each generated composite did not follow a consistent pattern for each of the blended-filled composites. The C70R20 blend, which corresponds to 70 and 20 phr of N772 and RHS respectively, yielded 97.49% bound rubber, the maximum of the developed blends. All composites indicated a good BRC, with 74.06% being the smallest amount recorded.

As shown in Fig. 4b, the composite with a formulation of 70 phr N772 and 20 phr RHS which resulted in the highest BRC also had the highest crosslink density. Studies^44^ on the characteristics of rubber composites blended with marble sludge and RHS as fillers indicated that the crosslink densities of marble sludge/RHS/NR blend were between 1.636 to 3 × 10^4^ mol/cm^3^ and stated that at 60 phr filler loading, the crosslinking network ultimately led to enhanced properties due to the restrictions created to the absorbance of the toluene. In this study, however, there was no correlation between crosslink densities and loading levels, indicating that crosslink densities are a function of the specific blend ratios rather than the wholistic formulation value in phr. In addition, the results obtained for the BRC and crosslink density are not completely consistent with the findings from researchers^45^ who asserted that an increase in the bound rubber content of rubber compounds resulted in a commensurate drop in the crosslink density.

**Figure 5:**
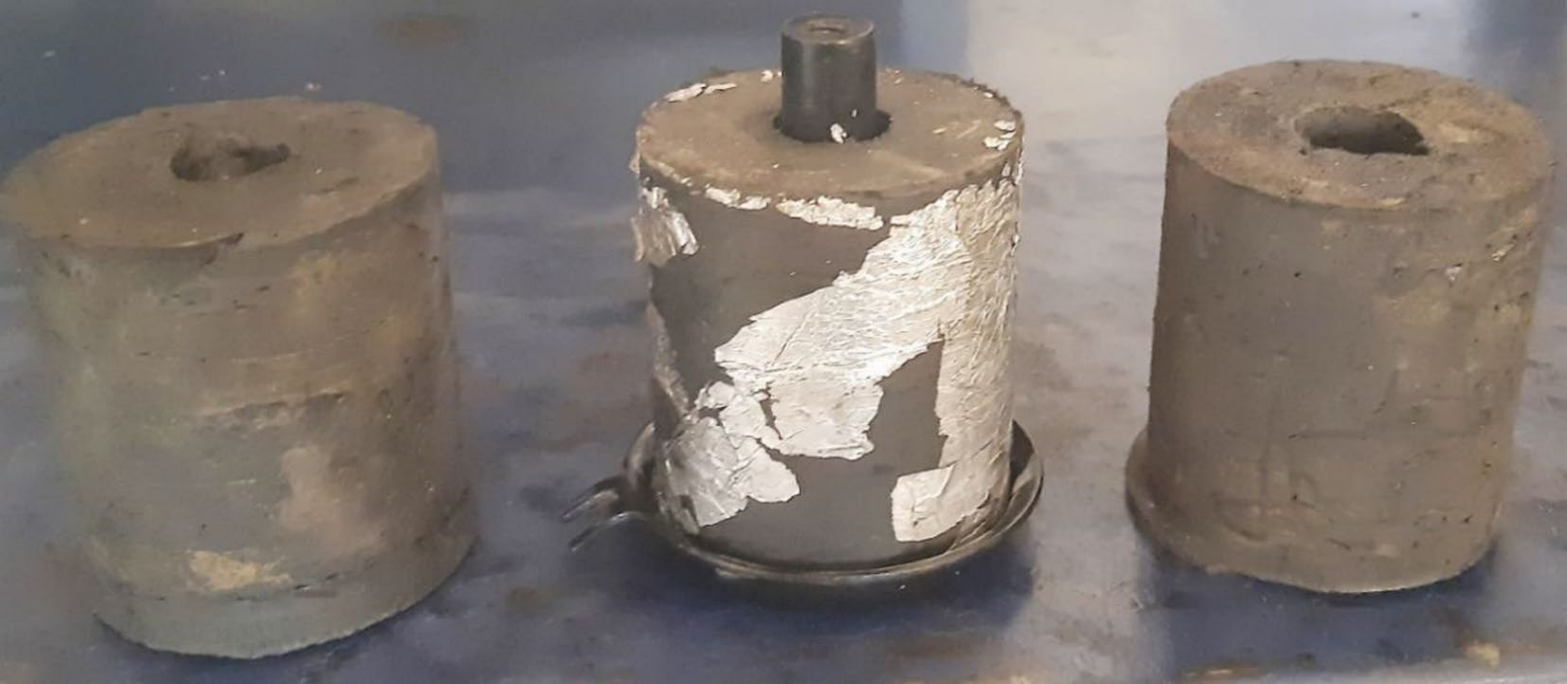
Developed RHS/N772 REM composites.

For the Shore A hardness, the measured values for all composites were within the ranges published by many studies^41,46,47^. A shore A hardness value between 45 to 70 has been reported to be the ideal hardness range for rubber engine mounts corresponding to an ideal filler loading range from 46 to 96 phr for high-load automotive rubber engine mounts^41,46,47^. Figure 4c shows the Shore A hardness of the RHS/N772 natural rubber composites. The average shore A hardness of the conventional REM sample utilised in the performance evaluation had a hardness value of 58. Figure 5 shows the image of the developed RHS/N772 blended REMs.

**Figure 6:**
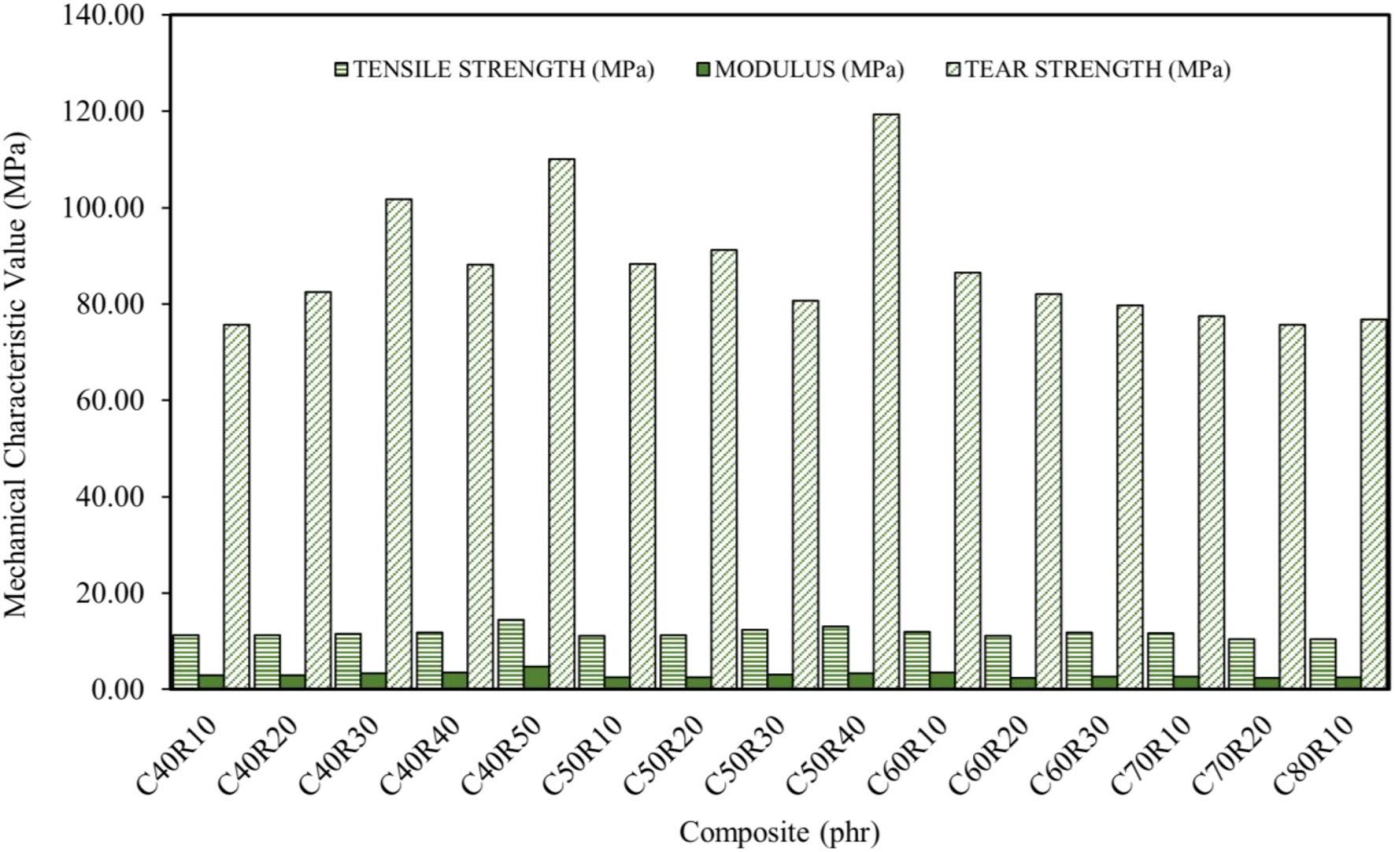
Tensile strength, modulus and tear strengths of blended filler composites.

Figure 6 shows the results for the tensile strength, tensile modulus, and tear strength of the RHS/N772 blended composites. Studies^44,46,48^, have shown that the increased modulus with increasing filler loading observed in the results was in part attributable to the effect of the rice husk silica particles in the composites. The excellent tensile strength and modulus of the composites can be attributed to the large specific surface area of the silica particles^48^. Furthermore, in their studies, Bonmee et al.^39^ posited that materials with smaller particle sizes had reduced tensile and tear strengths. The rigidity of natural rubber chains at high amounts of filler loading also affects the modulus behaviour of the composites.

According to a study^44^, the effect of blended fillers on modulus, tensile strength, and tear strength at high loading levels is mostly attributable to the reduced chain mobility of rubber molecules and the agglomeration of the fillers. In a similar study, the researchers^49^ observed tensile and tear strengths of 19.44 MPa and 87.5 MPa, respectively, for RHS composites with a filler loading of 40 phr. They also reported that the RHS-filled composites had superior tensile and tear properties compared to commercial silica-filled composites.

**Figure 7:**
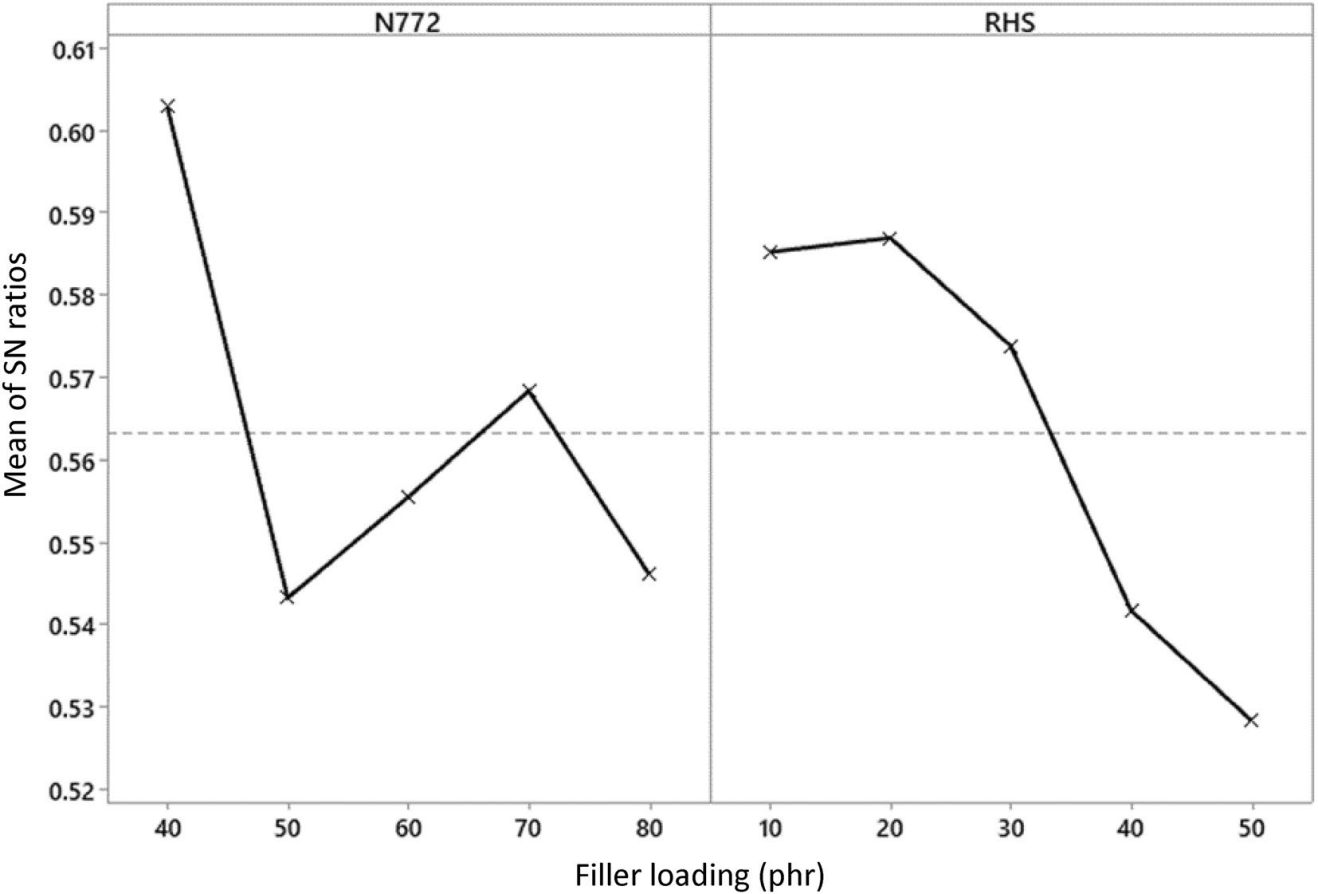
Main effect plots for the grey relational grade.

Researchers^30^ have established that blended fillers offer improved dispersion and filler-rubber interaction in composites, and that using fillers as a blend results in composites with superior properties when compared to composites developed from individual fillers. Grey relational analysis (GRA) was adopted to reduce the multi-response parameters to a single response, and the effect of the factors was utilised to determine the most viable alternative. According to the main effects plot in Fig. 7, the optimal filler blend level consisted of 40 phr of N722 matching level 1 and 20 phr of RHS matching level 2.

Many studies^46,50–52^ have also reported outstanding mechanical characteristics for rubber composites with filler loading levels between 50 and 70. A study^52^ combined RHS with commercial silica to achieve optimal mechanical properties, adopting a filler blend ratio of 20/30 phr for RHS and commercial silica, respectively. Similarly, in another study^50^, it was observed that the Silica blended with carbon black (SiO_2_/CB) ratio of 20/50 phr yielded the most exceptional characteristics when silica was blended with carbon black. The findings from implementing the GRA to determine the optimal filler combination resulted in a BRC of 90.59%, a crosslink density of 3.78 mol/cm^3^, a compressive set of 6.18%, 50 shore A hardness, a tensile strength of 11.27 MPa and a tear strength of 82.50 MPa.

Several researchers^30,53^ have established that the formulation of rubber composites has a significant influence on the characteristics of the composites. Therefore, while filler loadings are crucial in rubber composites, they are not the only factors responsible for the properties exhibited by rubber composites, especially when deployed in vibration isolation, as evidenced by the correlation coefficient (R^2^) values of various responses for blended composites presented in Table 5. R-squared values for all properties assessed against filler loadings demonstrated that complex factors other than filler loadings account for a significant amount of the responses of the developed RHS/N772 rubber composites. Other variables that may strongly impact and control the responses of natural rubber composites include the type and loading level of coupling agents (in cases where silica is used), the type and loading level of accelerators and additives, the size of filler particles, the specific surface area of fillers, and curing factors (temperature, pressure, time). According to several studies^54^, the R-squared values confirm the complex behaviour of elastomeric-based composites for vibration-isolating systems as depicted by the R-squared values. In research, authors^55^ who obtained comparable low correlation coefficient values for nano clay–nitrile rubber composites, indicated that certain fits were superior to others for particular responses. Similarly, researchers^56^ in a study obtained low R^2^ values of percentage elongation (47.1% and 23.3%, respectively) for two distinct natural rubber composites and concluded that the model does not fully explain the variability, indicating that this response is dependent on other characteristics. The shore A hardness of the developed RHS/N772 engine mount material in this study yielded an R^2^ value of 71.79% translating to high predictive strength of the regression model for this response.

**Table 5:** Correlation coefficient values of different responses for the blended composites.

Response	Degree	R^2^ (%)	Regression Equation
Compression Set	2	31.29	6.55 + 0.055 X—0.0918 Y—0.00085 X^2^—0.00069 Y^2^ + 0.001800 XY
	3	32.22	6.1 + 0.13 X − 0.221 Y − 0.0021 X^2^ + 0.0042 Y^2^ + 0.001800 XY + 0.000007 X^3^− 0.000055 Y^3^
	4	32.40	− 15 + 1.5 X − 0.00 Y − 0.039 X^2^ − 0.0092 Y^2^ + 0.00180 XY + 0.00042 X^3^ + 0.00027 Y^3^ − 0.000002 X^4^ − 0.000003 Y^4^
Shore A Hardness	2	71.70	25.0 + 0.51 X + 0.163 Y − 0.00157 X^2^ − 0.00114 Y^2^ + 0.00750 XY
	3	71.79	− 21 + 3.0 X + 0.20 Y − 0.044 X^2^ − 0.0026 Y^2^ + 0.00750 XY + 0.000233 X^3^ + 0.000017 Y^3^
	4	72.15	− 14 + 4 X − 4.8 Y − 0.08 X^2^ + 0.299 Y^2^ + 0.00750 XY + 0.0006 X^3^ − 0.0073 Y^3^ − 0.000002 X^4^ + 0.000061 Y^4^
Tensile Strength	2	18.23	15.24 − 0.164 X + 0.0885 Y + 0.00142 X^2^ − 0.00048 Y^2^ − 0.00080 XY
	3	20.28	11.2 + 0.12 X − 0.098 Y − 0.0035 X^2^ + 0.0066 Y^2^ − 0.00080 XY + 0.000028 X^3^ − 0.000079 Y^3^
	4	25.31	− 38 + 4.1 X − 1.45 Y − 0.108 X^2^ + 0.0890 Y^2^ − 0.00080 XY + 0.00121 X^3^ − 0.00207 Y^3^ − 0.000005 X^4^ + 0.000017 Y^4^
Young Modulus	2	46.17	6.84 − 0.1670 X + 0.0800 Y + 0.001517 X^2^ − 0.000320 Y^2^ − 0.000962 XY
	3	47.48	12.4 − 0.439 X + 0.016 Y + 0.0062 X^2^ + 0.00213 Y^2^ − 0.000962 XY − 0.000026 X^3^ − 0.000027 Y^3^
	4	50.22	49.0 − 2.84 X − 0.521 Y + 0.069 X^2^ + 0.0348 Y^2^ − 0.000962 XY − 0.00074 X^3^ − 0.000818 Y^3^ + 0.000003 X^4^ + 0.000007 Y^4^
Elongation	2	55.35	10 + 16.31 X − 7.32 Y − 0.1470 X^2^ + 0.0477 Y^2^ + 0.0787 XY
	3	57.98	− 790 + 57.4 X − 2.9 Y − 0.854 X^2^ − 0.119 Y^2^ + 0.0787 XY + 0.00393 X^3^ + 0.00185 Y^3^
	4	59.91	− 5242 + 365 X + 22.4 Y − 8.9 X^2^ − 1.66 Y^2^ + 0.0787 XY + 0.095 X^3^ + 0.0393 Y^3^ − 0.000379 X^4^ − 0.000312 Y^4^
Tear Strength	2	20.22	133.3 − 2.37 X + 1.50 Y + 0.0204 X^2^ − 0.0157 Y^2^ − 0.0047 XY
	3	22.42	− 19 + 6.5 X − 0.36 Y − 0.132 X^2^ + 0.055 Y^2^ − 0.0047 XY + 0.00085 X^3^ − 0.00079 Y^3^
	4	29.40	− 2662 + 199 X − 11.3 Y − 5.15 X^2^ + 0.72 Y^2^ − 0.0047 XY + 0.0578 X^3^ − 0.0170 Y^3^ − 0.000237 X^4^ + 0.000135 Y^4^
Bound Rubber Content	2	17.78	78.9 + 0.283 X − 0.084 Y − 0.00060 X^2^ + 0.00393 Y^2^ − 0.00515 XY
	3	28.23	224 − 8.26 X + 2.00 Y + 0.146 X^2^ − 0.0754 Y^2^ − 0.00515 XY − 0.000817 X^3^ + 0.000881 Y^3^
	4	47.42	2459 − 170.0 X + 8.64 Y + 4.37 X^2^ − 0.480 Y^2^ − 0.00515 XY − 0.0487 X^3^ + 0.0107 Y^3^ + 0.000199 X^4^ − 0.000082 Y^4^
Crosslink Density	2	30.13	1.50 + 0.038 X + 0.0603 Y + 0.000094 X^2^ − 0.000111 Y^2^ − 0.001173 XY
	3	46.52	26.3 − 1.382 X + 0.305 Y + 0.0245 X^2^ − 0.00943 Y^2^ − 0.001173 XY − 0.000136 X^3^ + 0.000104 Y^3^
	4	48.61	96 − 6.52 X + 0.711 Y + 0.159 X^2^ − 0.0342 Y^2^ − 0.001173 XY − 0.00166 X^3^ + 0.00070 Y^3^ + 0.000006 X^4^ − 0.000005 Y^4^

X = N772 and Y = RHS.

**Figure 8:**
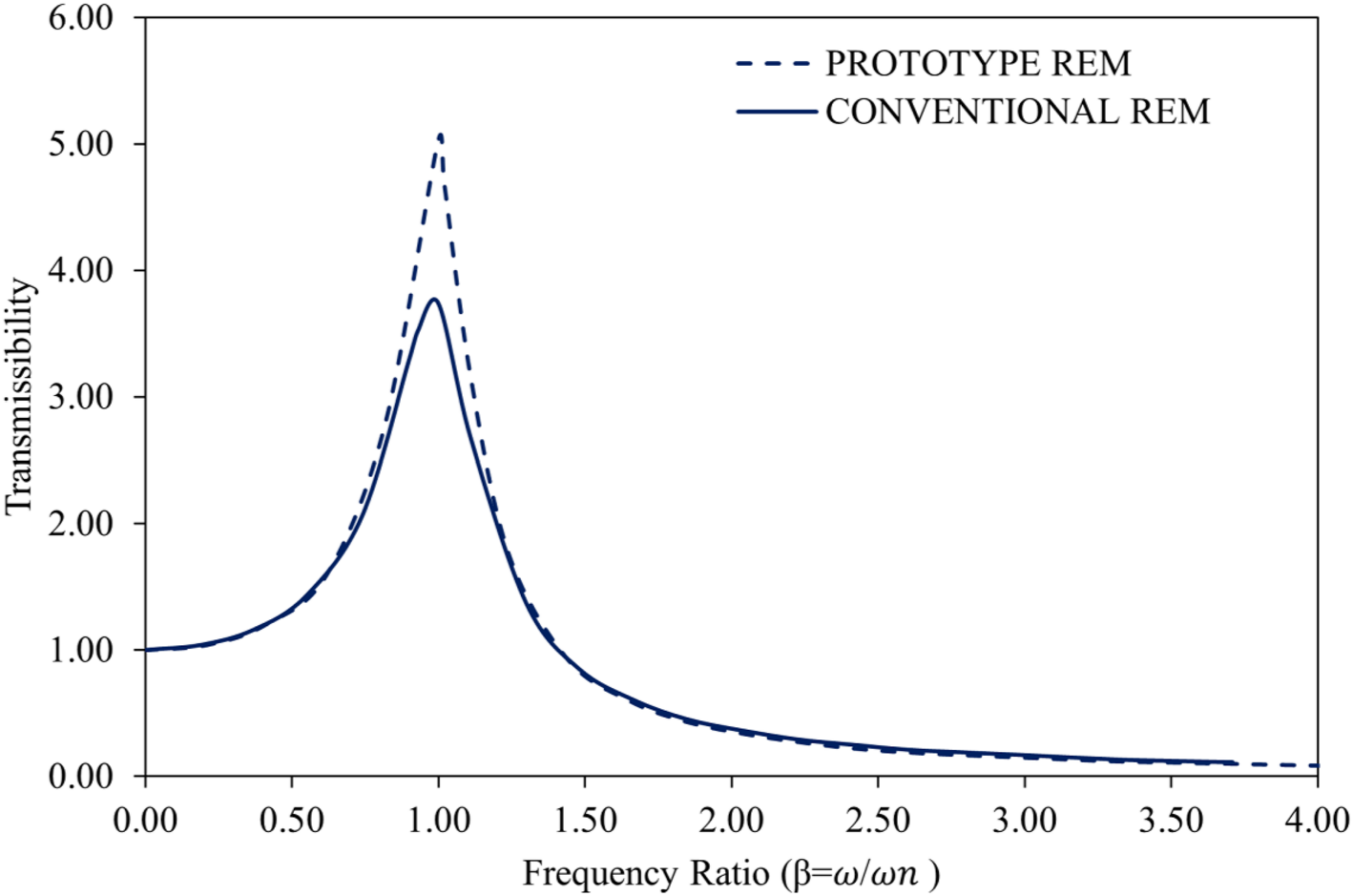
Transmissibility curve for the REMs.

The transmissibility curves for the conventional rubber engine mount and the produced RHS/N772 REM are shown in Fig. 8. For all REM systems at a frequency ratio of unity (β = 1), vibration is resonated and amplified to an infinite degree. For effective vibration isolation, the spring stiffness is always selected with care, and the frequency ratio is always tuned to be greater than 1.4. The dynamic spring stiffness is typically 1.20 to 1.40 times the static spring stiffness^57,58^. According to a study^59^, stiffness in rubber engine mounts typically occurs between 15 and 40 Hz and increases as frequency rises.

The resonance frequency of each REM is indicated by the sharp peaks in the transmissibility curve. The curve starts to abruptly fall at a frequency above the resonance frequency of the REM. It was noticed that the REMs started to isolate the system from the vibration effect with adequate damping characteristics as soon as the curve descended below the transmissibility at unity. Various studies have shown that the vibration isolator's cross-over point on a transmissibility curve is about 1.4 times the frequency of the isolator at resonance^58,60,61^. Since an increase in damping produced low transmissibility values in the isolation region, the vibration isolation becomes more effective with the descent of the transmissibility curve, which means that the reduction of vibration transmission occurs primarily at higher frequencies beyond the natural frequency. The transmissibility (at resonance) of the conventional and RHS/N772 REMs was 3.74 Hz and 5.03 Hz, respectively, following their respective natural frequencies of 26.96 Hz and 24.27 Hz. In an investigation, the researchers^59^ found a fundamental frequency of 27 Hz for their developed REM at which a very low dynamic stiffness value was attained, however, they stated that beyond that frequency the dynamic stiffness increased with increasing frequency. Although the RHS/N772 REM had high transmissibility at resonance, it provided greater damping properties and a lower transmissibility value in the isolation portion of the curve beyond resonance. Additionally, it was observed that tuning the engine mounts’ natural frequency to a lower value reduces transmissibility. Authors^62^ have asserted that engine mounts with six degrees of freedom experience a variety of natural frequencies for all axes and under different operating circumstances. They added that choosing and tuning natural frequencies for rubber engine mount systems always involves making compromises, particularly when efforts are made to alter the frequency from undesirable or typical excitation frequency ranges to lessen the high forces transmitted. While an eight-cylinder engine's frequency of disturbance is in the fourth order of the engines’ speed at a frequency range of 40 to 400 Hz for an engine speed of 600 to 6000 rpm as a four-cylinder engine, the frequency of fundamental disturbance for four-cylinder engines is at the second-order of the engines' speed with a frequency range between 20 and 200 Hz^62^.

**Figure 9:**
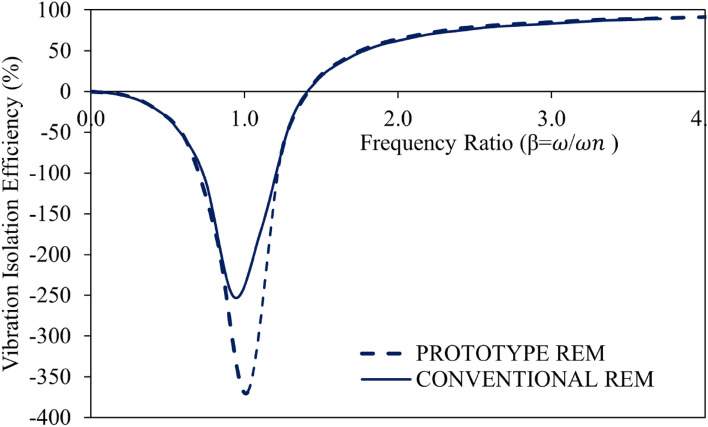
Vibration isolation efficiency curves for the REMs.

As established by several studies^58,61^, it is possible to achieve vibration isolation for all damping ratios if β > $\sqrt 2$. This is also true for the REMs examined in this study, as isolation was observed from the point where β is 1.4 on the transmissibility curve. The transmissibility curves for the tested REMs indicate that when the transmissibility is greater than or equal to unity, the vibration effect increases and smaller damping ratios are required to produce excellent vibration isolation. Sharp peaks that appeared in the transmissibility curve show vibration isolators that are only lightly dampened. Transmissibility curves with rounded or flattened peaks are typically connected to vibration isolators that have high damping ratios. The research results of several studies^37,46,63^ that low amplitude and high frequency require low damping and stiffness values while at low frequencies, typically less than 30 Hz, the rubber mount is expected to possess high stiffness and damping, are supported by the results of transmissibility curves obtained in this study. From the REMs’ vibration isolation efficiency curves shown in Fig. 9, it can be seen that the developed blended RHS/N772 composite can be used as an excellent vibration isolator due to its exceptional performance at all frequencies, according to the efficiency curves that emerge.

Results from vibration measurements taken on the test vehicle's engine head and chassis are shown in Table 6. The measurements were made at the engine head and chassis locations following the method used in a study^64^ for parameters of a similar type. 36 °C was the highest temperature recorded during testing at the engine mount locations.

**Table 6:** Vibration measurements on engine head and chassis.

Sample type	Location of probe	Engine speed (rpm)	Vibration velocity (mm/s)	Vibration acceleration (m/s^2^)	Displacement (mm)
Conventional REM	Engine head	0	4.20	8.90	0.150
		3000	29.60	34.00	0.147
		6000	73.00	126.80	1.481
	Chassis	0	2.50	4.90	0.060
		3000	11.30	19.70	0.090
		6000	11.37	20.40	0.074
Prototype REM	Engine head	0	9.20	11.70	0.208
		3000	32.00	40.10	0.440
		6000	44.60	141.10	1.792
	Chassis	0	4.40	6.30	0.164
		3000	13.20	20.80	0.143
		6000	14.50	23.70	0.028

The results obtained in this study for the vibration acceleration are comparable with those observations made in a study^65^ where the researchers reported a considerable increase in vibration acceleration values with increasing engine speed. Another study^64^ reported acceleration values of 154.22 m/s^2^, 304.30 m/s^2^ and 323.62 m/s^2^ at the engine head and 76.49 m/s^2^, 120.22 m/s^2^ and 138.27 m/s^2^ at the mount location for natural rubber engine mounts at engine speeds of 750, 1300 and 1800 rpm respectively. However, the researchers^64^ claimed that using butyl rubber in place of natural rubber led to higher acceleration values which they reported to be 129.09 m/s^2^, 223.49 m/s^2^ and 243.20 m/s^2^ at the engine head and 56.88 m/s^2^, 69.63 m/s^2^ and 110.82 m/s^2^ at the engine mount location for engine speeds of 750, 1300 and 1800 rpm respectively. When these values are compared to the acceleration values found in this study, it becomes clear that the RHS/N772 REM and conventional REM vibration acceleration values are significantly lower than those reported in a similar study^64^, especially when the speeds they tested for are taken into account. The ISO 10,816–1/10,816–6 standard's acceptable vibration limits for reciprocating engines^66,67^ were slightly exceeded at higher speeds by the samples' vibration velocity readings at the chassis. However, this slight change might not be caused by the engine mounts, but rather, in large part, by the condition of the engine's rotating and reciprocating parts. There is not much difference between the RHS/N772 REM and conventional rubber engine mount's chassis vibration measurements.

## Conclusion

In this study, rice husk silica (RHS) and carbon black N772 grade were blended and employed as fillers in natural rubber for vibration isolation applications. Results obtained showed good properties. The composite with a formulation of 70 phr N772 and 20 phr RHS which resulted in the highest BRC had the highest crosslink density, however, there was no correlation between crosslink densities and the filler loading levels, indicating that crosslink densities are a function of the specific blend ratios rather than the wholistic formulation value in phr. Shore A hardness values for the composites at different filler loading levels conformed with the ideal hardness range for engine mounts. The effect of blended fillers on modulus, tensile strength, and tear strength at high loading levels is attributable to the reduced chain mobility of rubber molecules and the agglomeration of the fillers at such loading levels. The rubber composites with combined filler loading levels between 50 to 70 phr exhibited favourable mechanical properties. Using Grey Relational Analysis, it was found that the blend of 40 phr of N772 and 20 phr of RHS is the most suited blend formulation for REM applications. Blending the fillers at different ratios greatly enhanced the mechanical and physical parameters of the composites. The resulting optimum blend can serve as an outstanding REM material by maintaining and offering a good compromise between static deflection and vibration isolation, based on the results obtained from this study. In addition to being a practical substitute and cost-effective filler material for use in the rubber industry, the use of RHS also helps to mitigate the environmental issues associated with the careless burning of rice husks in landfills and the use of non-sustainable fillers in the production of composite materials.

## Data Availability

The datasets used and/or analysed during the current study available from the corresponding author on reasonable request.
